# Yearly attained adherence to Mediterranean diet and incidence of diabetes in a large randomized trial

**DOI:** 10.1186/s12933-023-01994-2

**Published:** 2023-09-29

**Authors:** Miguel A. Martínez-González, Pedro Montero, Miguel Ruiz-Canela, Estefanía Toledo, Ramón Estruch, Enrique Gómez-Gracia, Jun Li, Emilio Ros, Fernando Arós, Alvaro Hernáez, Dolores Corella, Miquel Fiol, José Lapetra, Lluis Serra-Majem, Xavier Pintó, Montse Cofán, José V. Sorlí, Nancy Babio, Yolanda F. Márquez-Sandoval, Olga Castañer, Jordi Salas-Salvadó

**Affiliations:** 1 Instituto de Salud Carlos III, Centro de Investigacion Biomédica en Red Fisiopatología de la Obesidad y Nutrición (CIBERObn), Madrid, Spain; 2 IdiSNA (Instituto de Investigación Sanitaria de Navarra), Pamplona, Spain; 3 Department of Preventive Medicine and Public Health, University of Navarra, Pamplona, Spain; 4 Department of Nutrition, Harvard T.H. Chan School of Public Health, Boston, MA USA; 5 Department of Internal Medicine, Institut d’Investigacions Biomèdiques August Pi Sunyer (IDIBAPS), Hospital Clinic, University of Barcelona, Barcelona, Spain; 6 Department of Preventive Medicine, University of Malaga, Instituto de Investigación Biomédica de Málaga (IBIMA), Málaga, Spain; 7 Universitat Rovira i Virgili, Departament de Bioquimica i Biotecnologia, Unitat de Nutrició Humana (Grup ANUT-DSM). Institut d’Investigació Sanitària Pere Virgili, Reus, Spain; 8 Division of Preventive Medicine, Deparment of Medicine, Brigham and Women’s Hospital and Harvard Medical School, Boston, MA USA; 9 Lipid Clinic, Department of Endocrinology and Nutrition, August Pi i Sunyer Biomedical Research Institute (IDIBAPS), Hospital Clinic,, University of Barcelona, Barcelona, Spain; 10 Osakidetza Basque Health Service, Bioaraba Health Research Institute, Araba University Hospital, University of the Basque Country (UPV/EHU), Vitoria-Gasteiz, Spain; 11 Unit of Cardiovascular Risk and Nutrition, Institut Hospital del Mar d’Investigacions Mèdiques (IMIM), Barcelona, Spain; 12 Centre for Fertility and Health, Norwegian Institute of Public Health, Oslo, Norway; 13 Blanquerna School of Health Sciences, Universitat Ramon Llull, Barcelona, Spain; 14 Department of Preventive Medicine, University of Valencia, Valencia, Spain; 15 Platform for Clinical Trials, Instituto de Investigación Sanitaria Illes Balears (IdISBa), Hospital Universitario Son Espases, Palma de Mallorca, Spain; 16 Research Unit, Department of Family Medicine, Distrito Sanitario Atención Primaria Sevilla, Sevilla, Spain; 17 Nutrition Research Group, Research Institute of Biomedical and Health Sciences (IUIBS), University of Las Palmas de Gran Canaria, Las Palmas de Gran Canaria, Spain; 18 Lipids and Vascular Risk Unit, Internal Medicine, Hospital Universitario de Bellvitge, Hospitalet de Llobregat, Barcelona, Spain; 19 Departament de Bioquímica i Biotecnologia, Unitat de Nutrició Humana, Universitat Rovira i Virgili, Reus, Spain; 20 Cuerpo Académico UDG-CA-454 Alimentación y Nutrición en el Proceso de Salud-enfermedad, Centro Universitario de Ciencias de la Salud, Universidad de Guadalajara, Guadalajara, Mexico; 21 Centro de Investigacion Biomédica en Red de Epidemiología y Salud Pública (CIBERESP), Instituto de Salud Carlos III, Madrid, Spain

**Keywords:** Diabetes, Feeding trial, Nutritional epidemiology, Olive oil, Monounsaturated fats, Dietary assessment tools

## Abstract

**Background:**

Several large observational prospective studies have reported a protection by the traditional Mediterranean diet against type 2 diabetes, but none of them used yearly repeated measures of dietary intake. Repeated measurements of dietary intake are able to improve subject classification and to increase the quality of the assessed relationships in nutritional epidemiology. Beyond observational studies, randomized trials provide stronger causal evidence. In the context of a randomized trial of primary cardiovascular prevention, we assessed type 2 diabetes incidence according to yearly repeated measures of compliance with a nutritional intervention based on the traditional Mediterranean diet.

**Methods:**

PREDIMED (‘‘PREvención con DIeta MEDiterránea’’) was a Spanish trial including 7447 men and women at high cardiovascular risk. We assessed 3541 participants initially free of diabetes and originally randomized to 1 of 3 diets: low-fat diet (n = 1147, control group), Mediterranean diet supplemented with extra virgin olive (n = 1154) or Mediterranean diet supplemented with mixed nuts (n = 1240). As exposure we used actual adherence to Mediterranean diet (cumulative average), yearly assessed with the Mediterranean Diet Adherence Screener (scoring 0 to 14 points), and repeated up to 8 times (baseline and 7 consecutive follow-up years). This score was categorized into four groups: < 8, 8–< 10, 10– < 12, and 12–14 points. The outcome was new-onset type 2 diabetes.

**Results:**

Multivariable-adjusted hazard ratios from time-varying Cox models were 0.80 (95% confidence interval, 0.70–0.92) per + 2 points in Mediterranean Diet Adherence Screener (linear trend p = .001), and 0.46 (0.25–0.83) for the highest (12–14 points) versus the lowest (< 8) adherence. This inverse association was maintained after additionally adjusting for the randomized arm. Age- and sex-adjusted analysis of a validated plasma metabolomic signature of the Mediterranean Diet Adherence Screener (constituted of 67 metabolites) in a subset of 889 participants also supported these results.

**Conclusions:**

Dietary intervention trials should quantify actual dietary adherence throughout the trial period to enhance the benefits and to assist results interpretation. A rapid dietary assessment tool, yearly repeated as a screener, was able to capture a strong inverse linear relationship between Mediterranean diet and type 2 diabetes.

*Trial registration* ISRCTN35739639

**Supplementary Information:**

The online version contains supplementary material available at 10.1186/s12933-023-01994-2.

## Background

Diabetes prevention represents an uppermost priority for public health, with an expected global number of 643 million of persons with diabetes by 2030 [1]. Dietary habits are powerful determinants of diabetes risk [2]. Observational studies consistently reported inverse associations between the traditional Mediterranean diet (MedDiet) [3] and type 2 diabetes [4–6]. In particular, the MedDiet and the Mediterranean lifestyle are associated with lower frequency of metabolic syndrome and reduced all-cause mortality in Spain [7]. Even a modest increase in adherence to the MedDiet is linked to a decreased incidence of type 2 diabetes [8]. In addition to other mechanisms, the MedDiet has been reported to reduce advanced glycation end-products [9, 10]. In one center of the ‘‘Prevención con Dieta Mediterránea’’ (PREDIMED) trial, nut consumption, a component of the MedDiet pattern, was reported to be associated with a shift of the lipoprotein subfraction profile to a less atherogenic pattern, as well as with lower circulating concentrations of branched-chain aminoacids and decreased insulin resistance [11].

Furthermore, two randomized trials supported these benefits for the MedDiet, with 40% relative risk reduction for a MedDiet supplemented with extra-virgin olive oil (EVOO) [12] and increased remission rates with a low-carbohydrate MedDiet [13]. A much clearer understanding of these benefits can be attained by examining the actual compliance of participants in preventive trials, given that dietary interventions usually face suboptimal compliance and their results might not be generalizable to those following the same diet, but with a different compliance. The 14-item Mediterranean Adherence Screener (MEDAS) is a rapid assessment tool which validly appraises conformity to the MedDiet [14] and has been used and recommended in different countries [15–18]. This sort of dietary tools are useful in the context of a trial, because they can be repeated at different time intervals to assess dietary changes and provide immediate feedback to participants. The frequent repetition of assessments is a key methodological point, because repeated dietary measurements capture changes over time, thus increasing the validity and robustness of diet-disease associations [19]. We assessed the effect of adherence to the MedDiet on diabetes incidence in a large randomized trial using yearly repeated measurements.

## Methods

### Study design

PREDIMED is a primary cardiovascular prevention trial, whose methods were previously described [20, 21]. Briefly, participants were randomly allocated to one of three interventions in a 1:1:1 ratio: Mediterranean diet supplemented with EVOO (MedDiet + EVOO), Mediterranean diet supplemented with mixed nuts (MedDiet + nuts), or a control diet (low-fat diet). Intention-to-treat effects on type 2 diabetes incidence were previously reported [12], but that report did not consider actual compliance. Current recommendations for pragmatic trials encourage the assessment of actual compliance [22].

### Setting & participants

Randomized participants were 7447 men (55 to 80 years) or women (60 to 80 years) initially free of cardiovascular disease at baseline who were at high cardiovascular risk [21]. For this paper, only participants without type 2 diabetes at baseline and with assessment of glycemic control during the trial (n = 3541) were considered (1154 in MedDiet + EVOO group, 1240 in MedDiet + nuts group, 1147 in control diet). Participants in the three arms had personal interviews with a dietitian and small group meetings every 3 months to receive repeated education on the allocated diet, with practical advices on how to upgrade MedDiet (or low-fat diet) including recipes, seasonal food descriptions, shopping lists, meal plans, and menus. The MEDAS score, ranging from 0 (minimum) to 14 (maximum adherence) [14–18] of each participant was annually recorded in face-to-face interviews by the dietitians [21, 23].

### Mediterranean diet assessment

For each item in the MEDAS score, one point was awarded, briefly: preference of olive oil as the main culinary source of fat; preference of white meat over red/processed meat; daily consumption of olive oil (≥ 4 servings/d), vegetables (≥ 2 servings/d), fruit (≥ 3 pieces/d), red meat (< 1 serving/d), butter or margarine or cream (< 1 serving/d), and carbonated or sugar-sweetened beverages (< 1 cup/d); and weekly consumption of wine (≥ 7 cups/wk), pulses (≥ 3 servings/wk), fish/seafood (≥ 3 servings/wk), tree nuts (≥ 3 servings/wk), commercial pastry (< 2 servings/wk), and `sofrito´ (a sauce of tomato, garlic, onion or leeks sauteed in olive oil; ≥ 2 times/wk).

### Ascertainment of type 2 diabetes

The American Diabetes Association criteria were used to adjudicate new-onset cases of type 2 diabetes during follow-up. These criteria are: HbA1c ≥ 6.5% by a certified and standardized method according to the Diabetes Control and Complication trial, OR fasting (at least 8 h) plasma glucose ≥ 126 mg/dL (7 mmol/L), OR 2 h plasma glucose ≥ 200 mg/dL (11.1 mmol/L) during a standardized (75 g) oral glucose tolerance test, OR classic symptoms of hyperglycemia or hyperglycemic crisis with a random plasma glucose ≥ 200 mg/dL (11.1 mmol/L). In the absence of unequivocal hyperglycemia, diagnosis using these criteria requires two abnormal test results from the same sample or in two separate test samples [24]. Medical doctors, blinded to random allocations, reviewed patient medical records on a yearly basis and submitted potential type 2 diabetes cases to the Clinical Event Adjudication Committee of PREDIMED. This Committee confirmed incident cases blindly to the intervention groups. Only cases occurring during the trial’s intervention period (June 2003 to December 2010) were included in statistical analyses [21].

### Covariates

Other covariates collected in the PREDIMED trial have been previously described [20, 21]. Briefly, trained personnel measured blood pressure (in triplicate) and collected sociodemographic and lifestyle variables, including physical activity, using validated questionnaires [21]. Total energy intake was derived from a validated 137-item FFQ repeated yearly. We adjusted for the cumulative average of total energy intake at each yearly visit, and not only for baseline values.

### Statistical methods

The association between upgraded adherence to the MedDiet (using yearly cumulative averages) and type 2 diabetes was analyzed with multivariable time-varying Cox models. In these time-varying Cox models, the values of the variable that changed over time was the yearly cumulative average up to the current visit of each patient. For that aim, we time split the dataset into multiple rows per participant based on each visit (please see a very small synthetic example to mimic the data in the Supplementary material). Robust variance estimators were used to account for potential intra-cluster correlations among members of the same household or the same clinic in a small subset of participants. The main exposure were cumulative averages of MEDAS score, calculated as the mean of all time points up to that follow-up visit, including the baseline assessment and up to 7-year follow-up (i.e., 8 time points). Participants with missing values for MEDAS at any time point were assigned the mean of the score between the previous and posterior visit. Hazard Ratios (HRs) were calculated for the three categories of cumulative averages of the MEDAS ( 8 to < 10, 10 to < 12, and ≥ 12 scores) using as reference category the group with lowest adherence (MEDAS < 8). An additional HR was calculated for each 2-point increment in the MEDAS score (roughly equivalent to its standard deviation), considered as a quantitative variable. To quantify a linear trend, we conducted a Wald test for linear trend by assigning the median intake within each of the four categories and modeling this as a continuous variable. Multivariate model 1 was adjusted for age, sex, smoking status (never/current/former), dyslipidemia, hypertension, total energy intake level (kcal/d), physical activity (quintiles), and education (primary/secondary/university). Multivariable model 2 was additionally adjusted for propensity scores derived from estimated probabilities of allocation in the trial [21]. As an ancillary analysis, we also adjusted for the randomized arm of the trial. All models were stratified by recruitment center.

Subgroup analyses by sex, age (< 70 or ≥ 70 years), trial arm, BMI (< 30 or ≥ 30 kg/m^2^), and smoking were conducted. Interaction cross product-terms with cumulative averages of MEDAS (quantitative variable) were assessed with likelihood ratio tests in fully-adjusted models. To account for multiple testing these p values were corrected for the false discovery rate (FDR), using the Simes method [25]. We applied the same method to correct for multiple testing in the between-group comparison of baseline characteristics, after ANOVA for quantitative variables or after chi squared for categorical variables.

Incidence of diabetes was plotted by joint classification according to average MEDAS scores from years 1 to 7 (< 10 or ≥ 10) and randomized arm (merging both MedDiet groups together, and, only in an ancillary analysis by comparing only MedDiet + EVOO versus control). For these plots, we used standardization with inverse probability weighting (IPW) to control for confounding. Weighting factors were the previously mentioned potential confounders.

We also run (only as a reference) a conventional Cox model using *baseline* MEDAS as exposure and adjusting for all baseline potential covariates.

In a subset of 889 participants, information on a validated plasma metabolomic signature of MEDAS, comprised of 67 metabolites, was available. This signature has been well validated in PREDIMED and other cohorts with a reported r = 0.31–0.37 in PREDIMED [26]. A linear regression model was used to assess the predicted values of MEDAS with this 67-metabolite signature as predictor. Subsequently, an age- and sex-adjusted Cox model was fitted to obtain the HRs for diabetes according to categories of the metabolomic signature of MEDAS and per 2 additional points in the signature score.

Two tailed P values < 0.05 were considered significant and 95% confidence intervals were always computed.

## Results

### Description of participants

Baseline characteristics according to baseline MEDAS scores are shown in Additional file 4, Table S1. Table 1 shows characteristics of participants classified into four categories according to the combination of their average MEDAS during the entire follow-up period (< 10 points or ≥ 10 points, averaging repeated measures from year 1 to 7, i.e., excluding baseline values) and their randomized group control or MedDiet group (both MedDiets were merged). In this case we only used two categories of MEDAS (instead of the 4 previously mentioned categories of time-dependent cumulative averages) to obtain a sufficient number of subjects in this joint classification. Groups with higher average MEDAS score (i.e. ≥ 10 points) had a greater proportion of men, higher physical activity and energy intake but lower adiposity indexes and blood triglycerides.

**Table 1:** Characteristics of the study population according to the cross classification by randomized intervention and actual upgraded compliance with MedDiet during the trial period (average MEDAS during years 1 to 7)

MEDAS score during follow-up, excluding baseline	Control group		Both Mediterranean diet groups merged		p value^e^
	MEDAS < 10	MEDAS ≥ 10	MEDAS < 10	MEDAS ≥ 10	
	n = 906	n = 241	n = 785	n = 1609	
Mean follow-up (years 1 to 7) score in MEDAS (SD)	8.3 (1.0)	10.6 (0.6)	8.9 (0.8)	11.4 (0.9)	
Mean age (SD), year	67.2 (6.2)	67.1 (5.8)	66.3 (6.2)	66.4 (5.9)	0.002
Female sex %	68.2%	53.1%	64.6%	58.5%	<0 .001
Mean BMI (SD), kg/m^2^	30.4 (3.7)	29.1 (3.1)	30.5 (3.7)	29.6 (3.5)	< 0.001
Mean waist circumference (SD), cm	100 (10)	98 (9.8)	102 (11)	99 (10)	<0 .001
Mean waist-to-height ratio (SD)	0.63 (0.06)	0.61 (0.06)	0.64 (0.07)	0.62 (0.06)	< 0.001
Current smoker %^a^	15.8%	15.8%	16.3%	15.8%	0.005
Former smoker %	18.5%	30.7%	20.9%	22.9%	
Marital status: % married	71.8%	84.2%	72.6%	78.9%	< 0.001
Mean education level (SD), years	3.7 (0.7)	3.6 (0.8)	3.6 (0.8)	3.6 (0.8)	0.11
Obesity^b^	51.3%	38.2%	53.0%	42.1%	< 0.001
Overweight^b^	96.1%	93.8%	94.9%	94.4%	0.27
Hypertension^c^	92.9%	89.6%	91.8%	91.6%	0.41
Dyslipidemia^d^	85.3%	81.7%	83.3%	85.8%	0.27
Fasting glucose (SD), mg/dL	104 (18)	101 (15)	105 (18)	100 (16)	<0 .001
Total cholesterol (SD), mg/dL	218 (36)	217 (35)	220 (35)	220 (34)	0.586
HDL cholesterol (SD), mg/dL	55 (13)	56 (14)	56 (13)	56 (14)	0.18
LDL cholesterol (SD), mg/dL	137 (33)	136 (28)	137 (29)	138 (31)	0.55
Triglycerides (SD), mg/dL	137 (62)	129 (62)	141.0 (86)	126 (57)	< 0.001
Mean leisure-time physical activity level (SD), MET min/d	204 (200)	268 (252)	187 (194)	263 (239)	< 0.001
Mean total energy intake level (SD), kcal/d	2194 (567)	2301 (547)	2273 (599)	2345 (572)	<0 .001

Characteristics are for participants without diabetes at baseline (n = 3541)

*SD* standard deviation, *MEDAS* Mediterranean Diet Adherence Screener, *BMI * body mass index, *HDL * high-density lipoprotein cholesterol, *LDL*  low-density lipoprotein cholesterol, *MedDiet*  Mediterranean diet, *MET*  metabolic equivalent

^a^Current smoker was defined as > 1 cigarette, cigar, or pipe per day. Former smoker was defined as no smoking for at least 1 y

^b^Overweight was defined as BMI ≥ 25 kg/m^2^ and obesity as BMI ≥ 30 kg/m^2^

^c^Hypertension was defined as Systolic blood pressure ≥ 140 mm Hg, diastolic blood pressure ≥ 90 mm Hg, or use of antihypertensive agents

^d^Dyslipidemia was defined as LDL cholesterol levels ≥ 4.14 mmol/L (≥ 158.30 mg/dL), HDL cholesterol levels < 1.03 mmol/L (< 39.77 mg/dL) in men or < 1.29 mmol/L (< 49.81 mg/dL) in women or use of lipid-lowering therapy

^e^Corrected for the false discovery rate using the Simes method

Median follow-up was 4.1 years (interquartile range, 2.5 to 5.7). As expected, participants randomly allocated the two MedDiets attained a significantly better upgrading in MEDAS than those in the control group (P < 0.01 for each yearly comparison). After year 1, average ± SD MEDAS was 10.1 ± 1.4 in the MedDiet + EVOO group, 10.2 ± 1.5 in the MedDiet + nuts group, and 8.7 ± 1.5 in the control group. During follow-up, a MEDAS score ≥ 10 was observed in 57.8% of the MedDiet + EVOO group, in 62.5% of the MedDiet + nuts group, and in only 23.2% of the control group (Additional file 1: Figure S1).

Changes in medications that may influence onset of diabetes, such as antihypertensive drugs, statins, corticoids, or estrogens, were distributed evenly among groups (Additional file 2: Figure S2).

### Outcome data

**Fig. 1:**
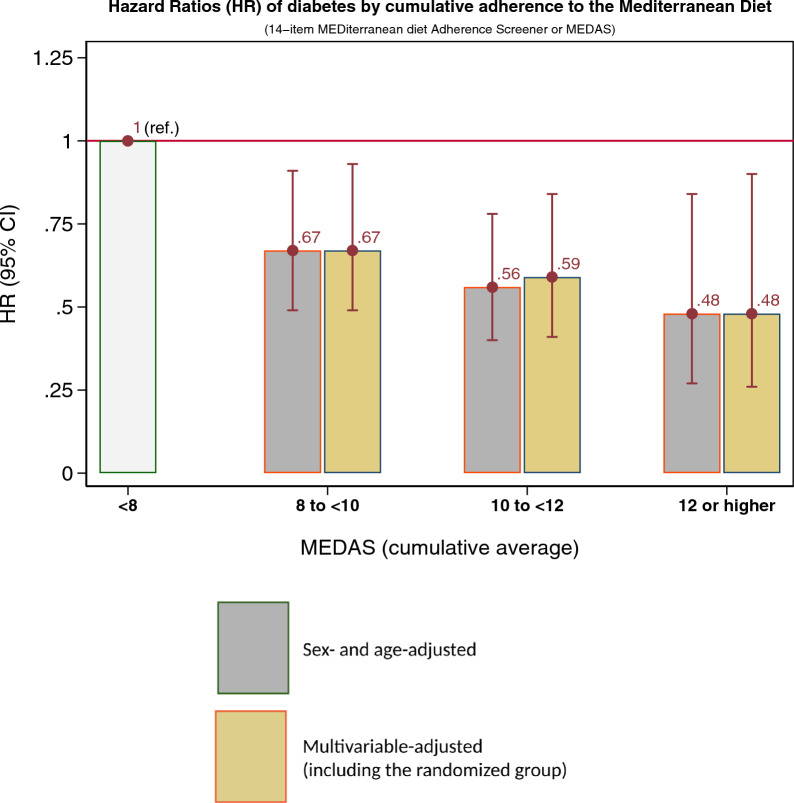
Hazard Ratios (HR) of developing type 2 diabetes according to cumulative adherence to the Mediterranean diet (0 to 14 score of the MEDiterranean diet Adherence Screener or MEDAS). Multivariable adjusted hazard ratios for successive categories of the MEDAS (cumulative averages using yearly repeated measures) according to estimates adjusted only for age and sex (grey bars) or according to estimates with multivariable adjustment for age, sex, baseline smoking status (never, current, or former smoker), fasting glucose level, prevalence of dyslipidemia (yes/no) and hypertension (yes/no), total energy intake level (kcal/d), physical activity level (metabolic equivalent of min/d), education level (primary education, secondary education, academic/graduate), propensity scores for group allocation and randomized group allocated (adjacent bars in sand color). All estimates were stratified by recruitment center, and robust SEs were used. *MEDAS* Mediterranean Diet Adherence Screener, *CI* confidence interval.

During follow-up, 273 incident cases of type 2 diabetes were blindly adjudicated by the Clinical Events Committee. Incidence rates per 1000 person-years by cumulative averages of MEDAS were 27.8 (< 8 points in MEDAS), 20.1 (8 to < 10), 16.1 (10 to < 12) and 13.8 (≥ 12 points in MEDAS), showing a monotonic descending trend as MEDAS increased (Additional file 4: Table S2). For each 2-point increment in MEDAS, the multivariable-adjusted analysis showed a 20% decrease in type 2 diabetes incidence (HR = 0.80; 95% CI, 0.70–0.92, p for linear trend = 0.001). Compared with participants in the lowest category (< 8 points in MEDAS), multivariable-adjusted HRs were 0.66 (95% CI 0.48 to 0.90) for the group scoring 8 to < 10 points, 0.56 (95% CI 0.40 to 0.79) for those scoring 10 to < 12 points, and 0.46 (95% CI 0.25 to 0.83) for those scoring ≥ 12 points, p for trend < 0.001 (Table 2 and Fig. 1). Additional adjustment for the randomized groups only slightly attenuated these results with HRs of 0.67 (95% CI 0.49–0.93) for 8 to < 10 points, 0.59 (95% CI 0.41–0.84) for 10 to < 12 points and 0.48 (95% CI 0.26–0.90) for ≥ 12 points, p for trend = 0.003 (Fig. 1). In the multivariable model adjusted for the randomized arm, a 2-point increment in MEDAS was associated with HR = 0.82 (95% CI 0.71–0.96). Therefore, higher adherence to the MedDiet linearly resulted in lower risk of diabetes, regardless of whether the participant was in the extra virgin olive oil or in the nuts intervention group.

**Table 2:** Hazard Ratios (95% confidence intervals [95% CI]) of type 2 diabetes according to upgraded cumulative average adherence to the Mediterranean diet (MEDAS^a^ score) during the trial

HR (95% CI)	Cumulative average of MEDAS^**a**^ In repeated measurements during follow-up^**b**^				Continuous
	< 8	8 to < 10	10 to < 12	> = 12	per 2-point increment
Crude model	1 (ref.)	0.67 (0.49–0.91)	0.56 (0.40–0.78)	0.48 (0.27–0.84)	0.82 (0.72–0.94)
Age- and sex-adjusted	1 (ref.)	0.66 (0.48–0.91)	0.55 (0.40–0.77)	0.46 (0.26–0.81)	0.81 (0.71–0.92)
Multivariate adjusted 1^**c**^	1 (ref.)	0.65 (0.47–0.89)	0.55 (0.39–0.77)	0.45 (0.25–0.81)	0.80 (0.70–0.91)
Multivariate adjusted 2^**d**^	1 (ref.)	0.66 (0.48–0.90)	0.56 (0.40–0.79)	0.46 (0.25–0.83)	0.80 (0.70–0.92)

^a^*MEDAS* mediterranean diet adherence screener

^b^Time-dependent Cox regression models with cumulative averages of repeated measures were used to assess the relative risk of type 2 diabetes by upgraded cumulative adherence to MedDiet (14-point score), estimating the HRs and 95% CIs. All models were stratified by recruitment center, and robust SEs were used

^c^Adjusted for age, sex, baseline smoking status (never, current, or former smoker), prevalence of dyslipidemia (yes/no) and hypertension (yes/no), total energy intake level (kcal/d), physical activity level (metabolic equivalent-min/d), educational level (primary education, secondary education, and academic/graduate)

^d^Additionally adjusted for propensity scores for being allocated to the different arms of the trial

These findings using cumulative averages were in contrast with the non-significant results found when only baseline MEDAS was assessed. The multivariable-adjusted HR per a 2-point difference in the baseline MEDAS was 0.95 (0.84 to 1.08, p = 0.44) and multivariable-adjusted HRs in categorical analyses were 0.91 (0.67 to 1.24) for 8 to < 10 points in baseline MEDAS; 0.87 (0.63 to 1.21) for 10 to < 12 points; and 0.64 (0.33 to 1.24) for ≥ 12 points.

Previously we developed and validated a plasma signature (a weighted combination of 67 metabolites) that well predicted MEDAS in both PREDIMED (Pearson correlation with MEDAS = 0.31–0.37, P < 0.001) and three independent US cohorts [26]. The age- and sex-adjusted Cox model showed HR = 0.59 (95% CI 0.39–0.89) for a metabolomic signature for MEDAS of 8 to < 10 and a HR = 0.49 (0.29–0.84) for a signature >  = 10 as compared to the metabolomic signature for MEDAS of < 8. A HR = 0.73 (0.54–0.99) was found per 2-point increment in the metabolomic signature for MEDAS.

In the time-dependent models using the whole sample (n = 3541), the absolute adjusted rate reduction was 14.4 cases prevented per 1000 persons-years, when comparing the highest (≥ 12 points) versus the lowest (< 8) category of MEDAS cumulative scores. Therefore, the estimated number needed to treat each year to prevent one case of type 2 diabetes was 69.

Subgroup analyses assessed per 2-point increments in cumulative MEDAS average during follow-up showed consistent inverse associations with type 2 diabetes. No significant interactions with stratifying variables were found, regardless of whether or not we corrected for the FDR (Additional file 3: Figure S3).

**Fig. 2:**
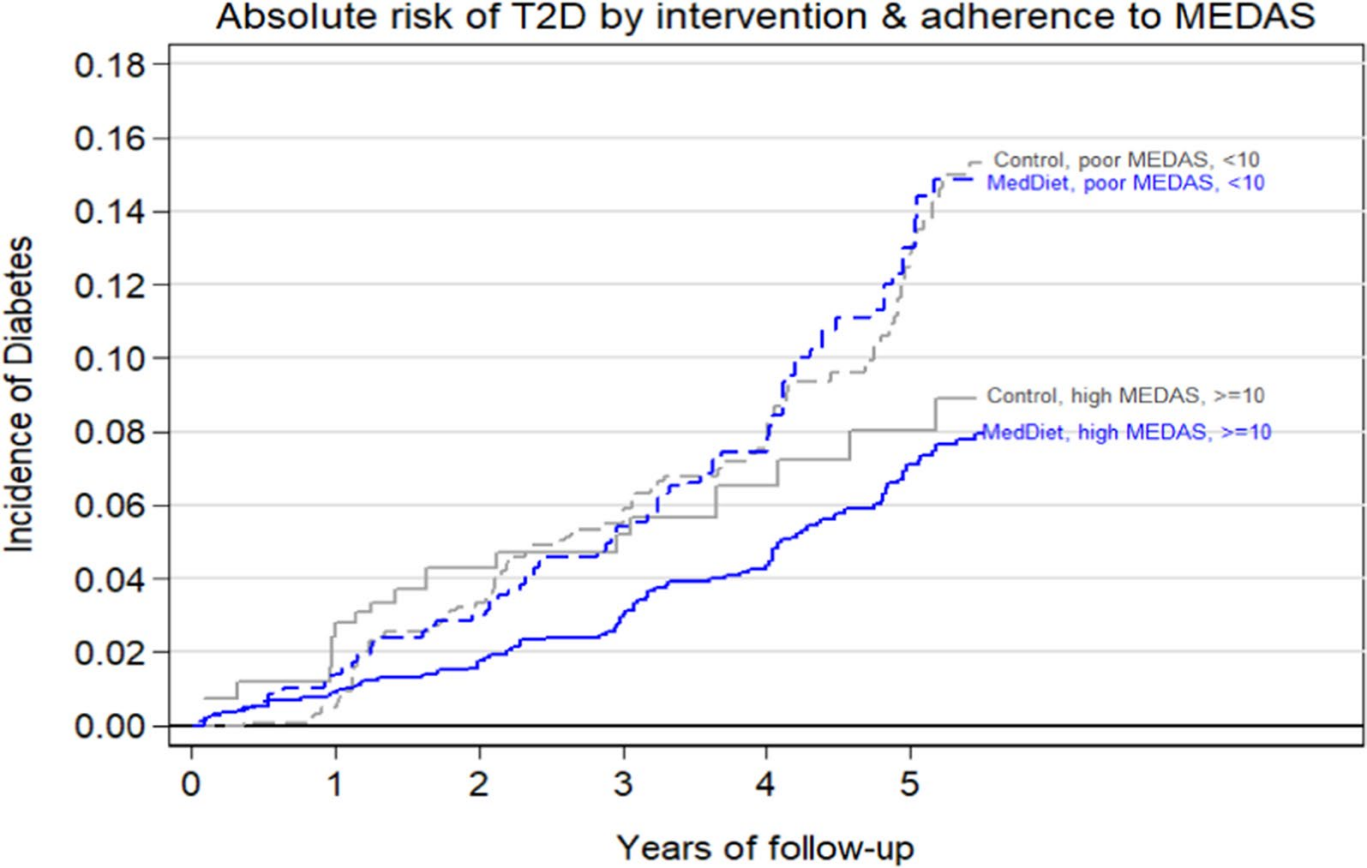
Cumulative incidence (Nelson-Aalen curves) of diabetes according to average MEDAS scores during years 1 to 7 and randomized intervention group. MEDAS scores are dichotomized, where ≥ 10 points are regarded as high adherence. Both intervention groups with Mediterranean Diet have been merged and they were compared against the control group. Adjusted for age, sex, baseline smoking status (never, current, or former smoker), prevalence of dyslipidemia (yes/no) and hypertension (yes/no), family history of CVD, total energy intake level (kcal/d), physical activity level (metabolic equivalent of min/d), education level (primary education, secondary education, and academic/graduate) and recruitment center using inverse probability weighting

Figure 2 shows the cumulative risk (Nelson Aalen estimates) of type 2 diabetes (T2D) according to the joint classification by average MEDAS scores attained beyond baseline, i.e., during years 1 to 7 of the trial (dichotomized at ≥ 10 points) and randomized arm (the 2 MedDiet groups merged together vs the control group). The intention-to-treat assessment of the effect of the randomized intervention observed a significant type 2 diabetes rate reduction when comparing the control group to MedDiet + VOO, but not to MedDiet + nuts [12]. Additional file 1 : Figure S1 shows cumulative risk of diabetes according to the joint exposure to MedDiet + VOO and average complicance.

The main apparent factor responsible for the diverging rates was adherence to MEDAS after 1-year follow-up. Only within the group of participants attaining highest MEDAS scores (≥ 10) after baseline there was a lower risk of type 2 diabetes among those belonging to the MedDiet intervention groups than in the control group (Additional file 2: Table S2). The respective rates of type 2 diabetes per 1000 person-years were 25.3 in the control group with low adherence, 24.5 in the intervention groups with low adherence, 18.3 in the control group with high adherence and 14.5 in the intervention groups with high adherence.

## Discussion

### Findings in context

A robust and strong protective effect against type 2 diabetes of actually upgraded adherence to the MedDiet was found in the PREDIMED trial among men and women at high cardiovascular risk. The use of yearly repeated measures of adherence to the diet in a large randomized trial represents the novelty of our study. Participants who exhibited a higher adherence during the trial –regardless of their randomized group– had a substantially lower risk of type 2 diabetes. Even after adjusting for the randomized group, a highly significant inverse linear dose–response relationship was noted for each 2-point improvement in MEDAS and higher adherence to the MedDiet linearly resulted in lower risk of diabetes, regardless of the provision of extra virgin olive oil or nuts in the intervention program. In addition, an objective multi-metabolite signature of adherence to the MedDiet measured in plasma [26] confirmed these results. These findings are consistent with those of most previous studies reporting benefits of the MedDiet against type 2 diabetes [8, 27–31], however, our results provide a more robust assessment of the adherence to the MedDiet by using yearly repeated measurements and accounting for cumulative exposure with a yearly updated information which was not been used in previous papers.

### The importance of capturing actual compliance with the intended diet

In nutritional epidemiology, repeated measurements of exposure are highly informative because they best represent long-term diet and minimize within-subject variation [19, 32]. As expected in large, long-term behavioural trials, even after random assignment to intervention, suboptimal compliance is usually observed. Close to one-third of participants assigned to the MedDiet arms did not attain a MEDAS ≥ 10, and 21% of participants in the control group followed the MedDiet on their own and reached a MEDAS ≥ 10 (Table 1), which was associated with reduced risk. Our previous intention-to-treat assessment found a statistically significant effect when comparing the control group to MedDiet + VOO, but no significance was found for MedDiet + nuts. Nevertheless, now we show that high compliers belonging to both MedDiet intervention groups received the greatest benefit regarding diabetes prevention. The advantage of intervention groups can be related to the likely higher consumption of freely provisioned EVOO and nuts, even after keeping constant the MEDAS score, particularly given the consistency, coherence and biological plausibility for their beneficial effect.

### Biological plausibility of our findings

The specific mechanisms by which the MedDiet reduces the risk of type 2 diabetes are not yet fully understood. However, some have been proposed. Excessive body weight, especially abdominal fat, substantially increases insulin resistance [33]. Better adherence to the MedDiet usually results in greater and more sustained weight loss as compared to other isocaloric, healthy plant-based diets [34, 35] and lower adiposity as supported by several randomized trials [36–38]. Furthermore, adherence to MEDAS was strongly inversely associated with abdominal obesity in our baseline assessment of the PREDIMED cohort [35]. The replacement of rapidly absorbed carbohydrates by monounsaturated fat from EVOO or polyunsaturated fat from tree nuts contributes to avoid insulin resistance [39]. Insulin sensitivity is also improved by the richness in fruits, vegetables, legumes, EVOO and whole grains in the MedDiet, delaying carbohydrate absortion and providing phenolic compounds with beneficial antioxidant and anti-inflammatory properties. Closer conformity to the MedDiet involves a higher dietary fiber intake and overall improvements in carbohydrate quality with substantial benefits against type 2 diabetes [40]. In fact, the MedDiet has been repeatedly reported to be associated with lower levels of inflammatory biomakers [41, 42], increased levels of adiponectin [43, 44] and reductions in advanced glycation end products [10].

### Effects of specific components of the Mediterranean diet

Individual components of the MedDiet have also been associated with lower risk of diabetes [45, 46]. Insulin sensitivity can be improved by phenolic compounds found in olive oil, especially EVOO, showing anti-inflammatory and anti-oxidative properties [47] and by whole grain consumption, but worsened by red meat, processed meat and sugar-sweetened beverage consumption. Tree nuts and peanuts have a low glycemic index and are rich in nutrients beneficial for diabetes risk, such as fiber, unsaturated fatty acids, magnesium, and phenolic compounds [5]. However, inverse associations of nut consumption with type 2 diabetes were not apparent in a meta-analysis of observational studies [48].

### Clinical usefulness of rapid dietary screeners

The MEDAS tool has attracted great interest in many Mediterranean and non-Mediterranean countries [14–18]. MEDAS allows rapid feedback to patients and certain goals may be easily set to improve adherence to the MedDiet, particularly in primary care settings. However, some potential disavantages of the MEDAS, particularly for its transferabilty to other countries have been reported [49, 50], including the difficulties to appraise the item for ‘‘sofrito’’, the absence of an upper limit in the item for wine, the restriction to pastries in the cereal group and the inability to capture total energy intake. Notwithstanding, in our present assessment and in many other instances the MEDAS showed validity for predicting hard clinical outcomes in the long-term. Our results show that by improving only 2 items (an easily attainable and practical goal), a relative reduction in type 2 diabetes by 20% can be achieved.

### Limitations and strengths

Limitations in our study include, first, that our participants were at high risk of cardiovascular disease and they belonged to a Mediterranean country, thus reducing generalizability. Nevertheless, external validity should take into account biological plausibility and not only representativeness in the merely statistical interpretation used for surveys. Secondly, even though we accounted for many potential confounders, a potential for residual confounding exist; however, previous intention-to-treat results were reasonably free of residual confounding and also showed strong benefits. Thirdly, a certain degree of measurement error using MEDAS may have occurred; however, the face-to-face interviews by expert and well-trained dietitians to collect the MEDAS questionnaires may miminize this risk; also, the use of cumulative averages is known to reduce measurement errors [19, 32]. Furthermore, the objective exposure assessment with a plasma metabolomic-based measurement corroborated our findings.

Strengths of our study included addressing the important issue of the assessment of the effect of actual compliance with a dietary intervention in a large randomized trial. Secondly, those variables which met the requirements as to be considered major potential confounders were appropriately controlled for in the statistical analyses (we did not adjust for intermediate links in the causal pathway, to avoid overadjustment). In any case, it was reassuring that the results did not materially change after a variety of multivariable adjustments. Thirdly, we used cumulative averages of diet scores, which accounted for previous dietary exposures and reduced measurement errors. Fourth, biomarkers of adherence in a large subset of participants were used to confirm the validity of results.

## Conclusions

Our study found that upgraded adherence to the Mediterranean dietary pattern, high in virgin olive oil, fruits, vegetables, legumes, whole grains, nuts, fish and seafood; with moderate consumption of alcohol; and low intake of red and processed meat, whole-fat dairy, sodas, and sweets reduced the incidence of type 2 diabetes in subjects at high cardiovascular risk. These findings represent a practical and affordable approach to reduce the growing population burden of diabetes.

### Supplementary Information

**Additional file 1: Figure S1.** Risk of type 2 diabetes according to the joint classification to the Mediterranean diet+extravirgin olive oil (EVOO) or control group and averaged attained cumulative adherence to the Mediterranean diet (MEDAS score) during years 1 to 7 of follow-up. Cumulative incidence (Nelson-Aalen curves) considering only 2 randomized groups: Mediterranean Diet+extravirgin olive oil (EVOO) or control group (the group allocated to Mediterranean Diet + nuts was excluded). MEDAS scores are dichotomized, where ≥10 points are regarded as high adherence. Adjusted for age, sex, baseline smoking status (never, current, or former smoker), prevalence of dyslipidemia (yes/no) and hypertension (yes/no), family history of CVD, total energy intake level (kcal/d), physical activity level (metabolic equivalent of min/d), education level (primary education, secondary education, and academic/graduate) and recruitment center using inverse probability weighting.

**Additional file 2 :Figure S2. ** Changes in medication according to subgroups defined by the joint combination of average cumulative adherence to the Mediterranean diet (MEDAS, with cut-off point=10) and randomized group. The statin group was included in the lipid-lowering group, but it is also presented separately. *MEDAS* Mediterranean diet adherence score. *HRT *hormone replacement therapy (only considering women)

**Additional file 3: Figure S3.** Subgroup analysis of multivariable adjusted hazard ratios for each 2-point increment in MEDAS (cumulative averages using yearly repeated measures). P-values for interaction were calculated for subgroups of sex, age, intervention allocation, body mass index (BMI) and smoking status. Adjusted for age, sex, baseline smoking status (never, current, or former smoker), fasting glucose level, prevalence of dyslipidemia (yes/no) and hypertension (yes/no), total energy intake level (kcal/d), physical activity level (metabolic equivalent of min/d), education level (primary education, secondary education, academic/graduate), and propensity scores. Stratified by recruitment center, and robust SEs were used.

**Additional file 4: Table S1.** Baseline characteristics of the study population^a^. **Table S2.** Incidence of diabetes after a median follow-up of 4.1 years in the PREDIMED trial according to the randomized group and to attained cumulative average adherence to the Mediterranean diet (MEDAS score) during follow-up (across years 1 to 7).

## Data Availability

We will be happy to provide access to the data supporting the results reported in this article (including data dictionaries), making possible the replication of the analyses used for the present article. Due to the restrictions imposed by the Informed Consent and the Institutional Review Board, bona fide investigators interested in analyzing the dataset used for the present article may submit a brief proposal and statistical analysis plan to the corresponding author (mamartinez@unav.es). Upon approval from the Predimed Steering Committee and Institutional Review Boards, the data will be made available to them using an onsite secure access data enclave.

## Abbreviations

CI: Confidence intervals
EVOO: Extra-virgin olive oil
HR: Hazard ratio
MEDAS: Mediterranean diet adherence screener
MedDiet: Mediterranean diet
PREDIMED: *PREvención con DIeta MEDiterránea* (Prevention with Mediterranean Diet)
