# Supplementary figures and images for: Yearly attained adherence to Mediterranean diet and incidence of diabetes in a large randomized trial
Source: Cardiovasc Diabetol. 2023 Sep 29;22:262. doi: 10.1186/s12933-023-01994-2 (PMC10542699; doi:10.1186/s12933-023-01994-2)

## Slide 1
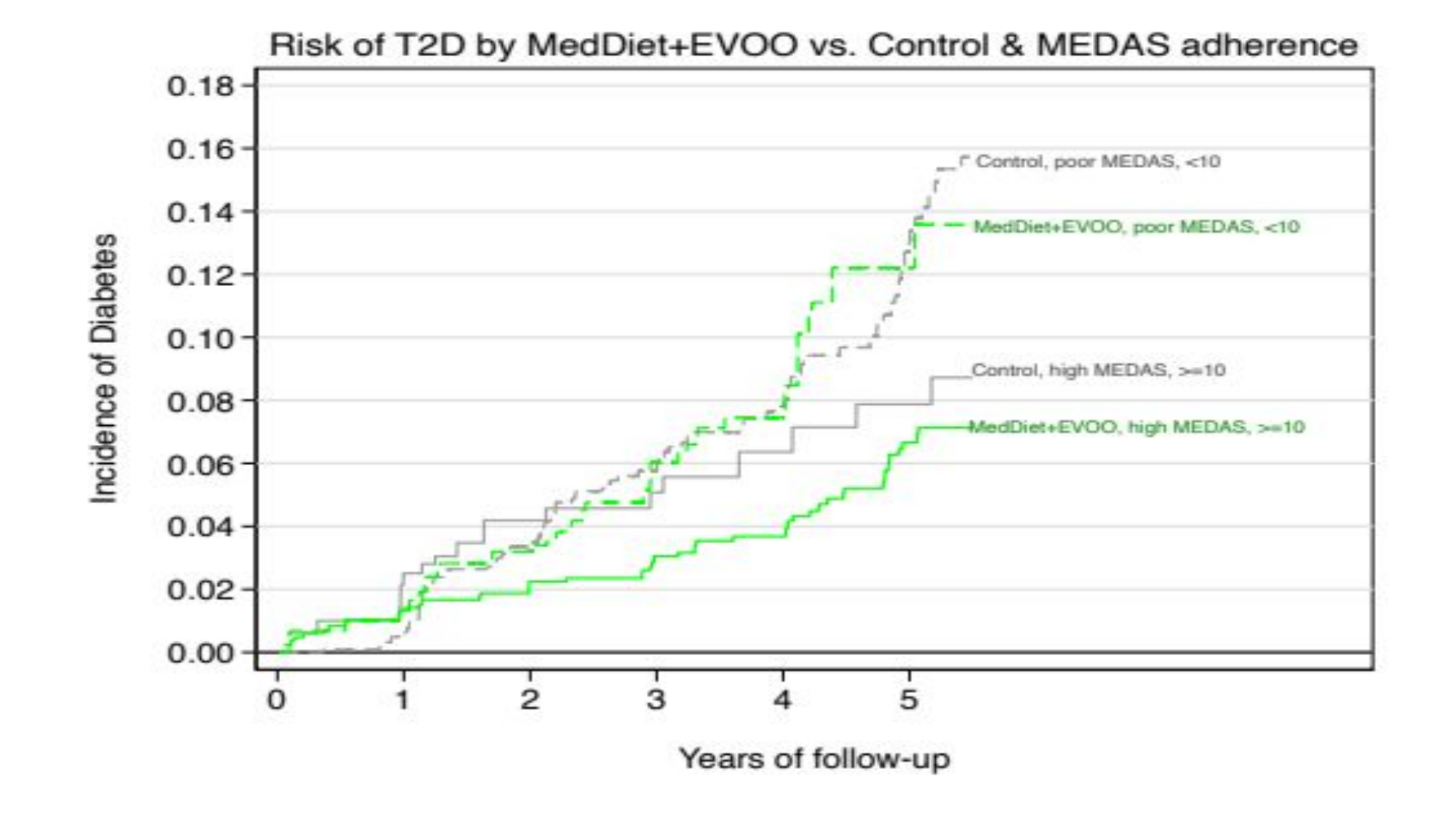

Supplement: Supplementary file 1 — Additional file 1: Figure S1. Risk of type 2 diabetes according to the joint classification to the Mediterranean diet+extravirgin olive oil (EVOO) or control group and averaged attained cumulative adherence to the Mediterranean diet (MEDAS score) during years 1 to 7 of follow-up. Cumulative incidence (Nelson-Aalen curves) considering only 2 randomized groups: Mediterranean Diet+extravirgin olive oil (EVOO) or control group (the group allocated to Mediterranean Diet + nuts was excluded). MEDAS scores are dichotomized, where ≥10 points are regarded as high adherence. Adjusted for age, sex, baseline smoking status (never, current, or former smoker), prevalence of dyslipidemia (yes/no) and hypertension (yes/no), family history of CVD, total energy intake level (kcal/d), physical activity level (metabolic equivalent of min/d), education level (primary education, secondary education, and academic/graduate) and recruitment center using inverse probability weighting. [file 12933_2023_1994_MOESM1_ESM.pptx]

## Slide 1
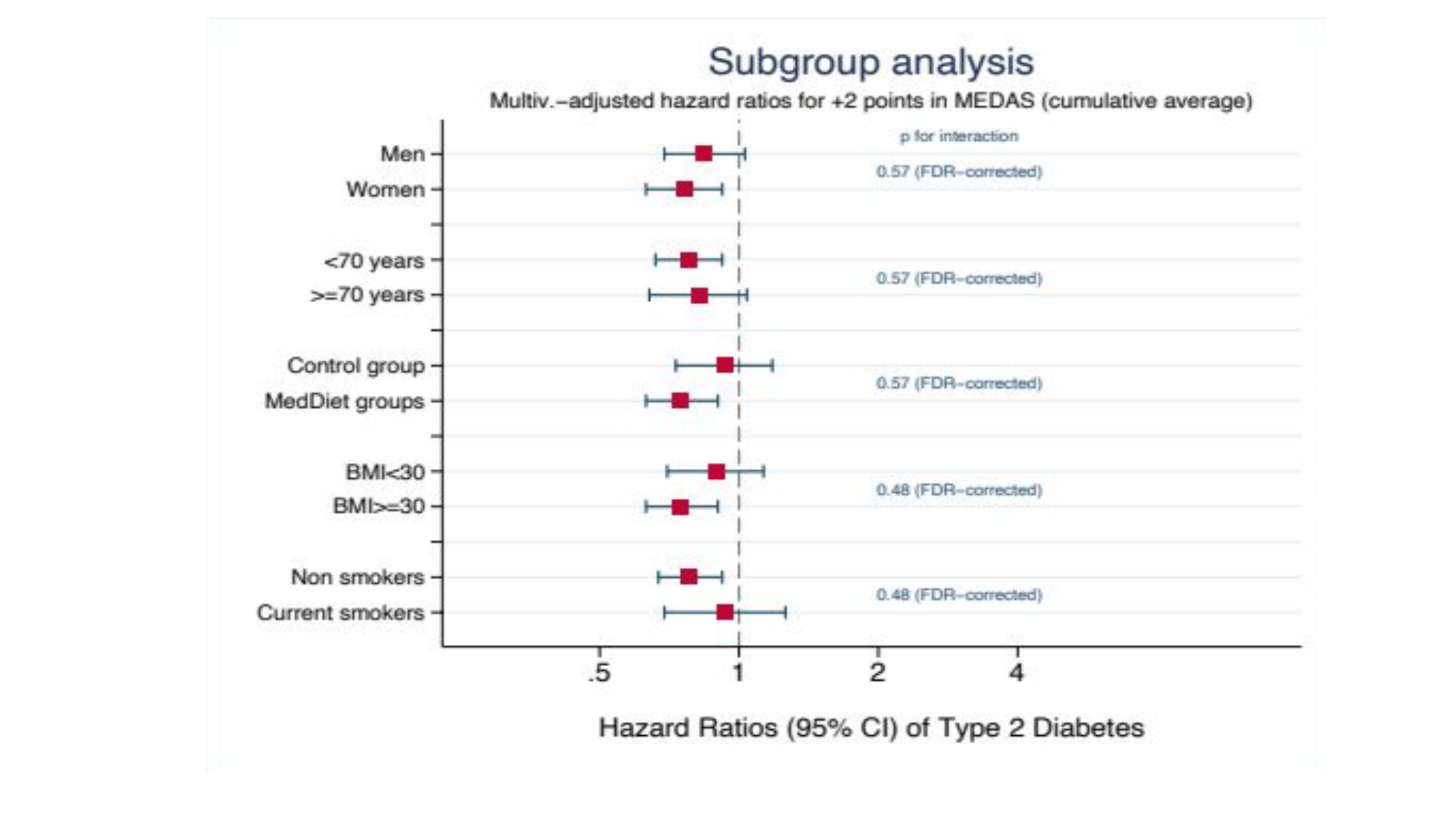

Supplement: Supplementary file 3 — Additional file 3: Figure S3. Subgroup analysis of multivariable adjusted hazard ratios for each 2-point increment in MEDAS (cumulative averages using yearly repeated measures). P-values for interaction were calculated for subgroups of sex, age, intervention allocation, body mass index (BMI) and smoking status. Adjusted for age, sex, baseline smoking status (never, current, or former smoker), fasting glucose level, prevalence of dyslipidemia (yes/no) and hypertension (yes/no), total energy intake level (kcal/d), physical activity level (metabolic equivalent of min/d), education level (primary education, secondary education, academic/graduate), and propensity scores. Stratified by recruitment center, and robust SEs were used. [file 12933_2023_1994_MOESM3_ESM.pptx]
