# Supplementary material for: Yearly attained adherence to Mediterranean diet and incidence of diabetes in a large randomized trial
Source: Cardiovasc Diabetol. 2023 Sep 29;22:262. doi: 10.1186/s12933-023-01994-2 (PMC10542699; doi:10.1186/s12933-023-01994-2)
Supplement: Supplementary file 4 — Additional file 4: Table S1. Baseline characteristics of the study populationa. Table S2. Incidence of diabetes after a median follow-up of 4.1 years in the PREDIMED trial according to the randomized group and to attained cumulative average adherence to the Mediterranean diet (MEDAS score) during follow-up (across years 1 to 7). [file 12933_2023_1994_MOESM4_ESM.docx]

**Additional file Table S1. Baseline characteristics of the study population ^a^**

|  | **Baseline Mediterranean diet adherence score (0 to 14)** | | | |  |
| --- | --- | --- | --- | --- | --- |
| Variable | 0 to 7 | 8 to 9 | 10 to 11 | 12 to 14 | p value^f^ |
| Participants | 943 | 1349 | 1039 | 210 |  |
| Mean age (SD), y | 66.6 (6.1) | 66.7 (6.0) | 66.7 (6.0) | 66.2 (5.7) | .76 |
| Female sex | 64.0 % | 62.2 % | 61.7 % | 52.9 % | .06 |
| Mean BMI (SD), kg/m² | 30.2 (3.7) | 30.2 (3.6) | 29.7 (3.6) | 29.4 (3.5) | .001 |
| Mean waist circumference (SD), cm | 100.3 (10.3) | 100.2 (10.4) | 98.8 (10.6) | 98.0 (11.7) | .001 |
| Mean waist-height ratio (SD) | 0.63 (0.06) | 0.63 (0.06) | 0.62 (0.06) | 0.61 (0.07) | <.001 |
| Tobacco use **^b^** |  |  |  |  | .008 |
| Never smoker | 63.9 % | 62.4 % | 60.6 % | 60.0% |  |
| Current smoker | 16.8 % | 17.4 % | 15.2 % | 12.4 % |  |
| Former smoker | 19.3 % | 20.2 % | 25.2 % | 27.6 % |  |
| Marital status: % married | 73.7 % | 75.9 % | 78.1 % | 78.6 % | .19 |
| Mean education level (SD), years | 3.96 (2.30) | 4.04 (2.36) | 4.13 (2.30) | 4.31 (2.52) | .23 |
| Obesity **^c^** | 49.6 % | 49.3 % | 42.2 % | 37.6 % | <.001 |
| Overweight **^c^** | 94.9 % | 95.3 % | 95 % | 92.4 % | .52 |
| Hypertension **^d^** | 92.0 % | 92.2 % | 91.5 % | 90.5 % | .87 |
| Dyslipidemia **^e^** | 85.2 % | 84.3 % | 85.3 % | 84.3 % | .87 |
| Fasting glucose (SD), mg/dL | 101.5 (17.4) | 103 (16.2) | 102.3 (17.9) | 101.4 (14.5) | .54 |
| Total cholesterol (SD), mg/dL | 222.7 (41.5) | 223.0 (39.9) | 224.4 (37.0) | 219.3 (38.8) | .22 |
| HDL cholesterol (SD), mg/dL | 53.3 (13.7) | 53.4 (13.1) | 54.7 (13.4) | 53.7 (14.1) | .57 |
| LDL cholesterol (SD), mg/dL | 138.4 (35.9) | 141.1 (36.1) | 143.4 (33.6) | 142.6 (31.9) | .22 |
| Triglycerides (SD), mg/dL | 140.6 (67.6) | 148.9 (75.7) | 133.1 (62.8) | 124.3 (51.3) | .17 |
| Leisure-time physical activity (SD), MET min/d | 207 (209) | 212 (201) | 262 (250) | 311 (251) | <.001 |
| Mean total energy intake level (SD), kcal/d | 2258 (616) | 2274 (579) | 2327 (565) | 2467 (523) | <.001 |

BMI = body mass index; HDL = high-density lipoprotein; LDL = low-density lipoprotein; MedDiet = Mediterranean diet; MET = metabolic equivalent.

**^a^** Values are numbers (percentages) unless otherwise indicated.

Characteristics are for all participants without diabetes (n = 3541).

**^b^** Current smoker was defined as >1 cigarette, cigar, or pipe per day. Former smoker was defined as no smoking for _1 y.

**^c^** Overweight was defined as BMI ≥25 kg/m2 and obesity as BMI ≥ 30 kg/m2.

**^d^** Systolic blood pressure ≥140 mm Hg, diastolic blood pressure ≥90 mm Hg, or use of antihypertensive agents.

**^e^** LDL cholesterol levels ≥4.14 mmol/L (≥158.30 mg/dL), HDL cholesterol levels ≥1.03 mmol/L (≥39.77 mg/dL) in men or ≥1.29 mmol/L (≥49.81 mg/dL) in women or use of lipid-lowering therapy.

**^f^** Corrected for the false discovery rate using the Simes method.

**Supplemental Table S2. Incidence of diabetes after a median follow-up of 4.1 years in the PREDIMED trial according to the randomized group and to attained cumulative average adherence to the Mediterranean diet (MEDAS score) during follow-up (across years 1 to 7)**

|  | Control group | | Both Mediterranean diet groups | | | |
| --- | --- | --- | --- | --- | --- | --- |
| **Average adherence to Medit. diet (MEDAS)** | <10 | ≥10 | <10 | | ≥10 | |
| Person-years | 3287 | 984 | 2898 | | 6977 | |
| New cases of diabetes, n | 83 | 18 | 71 | | 101 | |
| Crude rates per 1000 person-years (95% CI) | 25.3 (20.4-31.3) | 18.3 (11.5-29.0) | 24.5 (19.4-30.9) | | 14.5 (11.9-17.6) | |
| Multivariable-adjusted HR (95% CI) | 1 (ref.) | 0.65 (0.38-1.09) | 0.98 (0.70-1.37) | | 0.57 (0.42-0.77) | |
|  |  | |  | | | |
| **Average adherence to Medit. diet (MEDAS)** | | | <10 | | ≥10 | |
| Intervention arm of the trial | | | MedDiet+nuts | MedDiet+EVOO | MedDiet+nuts | MedDiet+EVOO |
| Person-years | | | 1350 | 1548 | 984 | 3449 |
| New cases of diabetes, n | | | 35 | 36 | 18 | 44 |
| Crude rates per 1000 person-years (95% CI) | | | 25.9 (18.6-36.1) | 23.2 (16.8-32.2) | 16.2 (12.5-20.9) | 12.8 (9.5-17.1) |
| Multivariable-adjusted HR (95% CI) **^a^** | | | 0.99 (0.66-1.50) | 0.97 (0.65-1.45) | 0.62 (0.44-0.88) | 0.51 (0.35-0.74) |

CI: Confidence interval; MEDAS: Mediterranean Diet Adherence Screener

**^a^** Reference category: control group and poor adherence.

**STATA codes with some explanations**

stset date_end, fail(diab_inc==1) enter(date_randomiz) id(id)

forval f=1/7 { **// f1 to f7: dates of follow-up visits**

replace f`f'=999999 if f`f'==. **// missing dates moved beyond the final date**

}

forval n=1/7 {

stsplit band`n', at(0) after(f`n')

replace band`n'=. if f`n'==.

}

g year=0 if band1<0 | band1==.

forval x=1/7 { **// events >7 y are related to cum. exposure up to year 7**

replace year=`x' if band`x'==0

}

g p14cum=.

foreach x of numlist 0/7 {

egen temp=rowmean(P14_0-P14_`x') **// P14_0 to P14_7 are the repeated MEDAS**

replace p14cum=temp if year==`x'

drop temp

}

***(something similar is done for the other time-dependent covariates, e.g. total energy intake)**

g p14cum2=p14cum/2 **// per +2 points in the 0-14 cum average of MEDAS**

stcox p14cum2 $COV $option **// $COV & $option are vectors (global macros) for covariates and options**

**Small synthetic dataset to mimic the aspect of the time-varying covariate**

| **id** | **year** | **P14_0** | **P14_1** | **P14_2** | **P14_3** | **P14_4** | **P14_5** | **P14_6** | **P14_7** | **p14cum (time-varying)** |
| --- | --- | --- | --- | --- | --- | --- | --- | --- | --- | --- |
| **1** | **0** | **12** | **9** | **9** |  |  | **9** |  |  | **12** |
| **1** | **1** | **12** | **9** | **9** |  |  | **9** |  |  | **10.5** |
| **1** | **2** | **12** | **9** | **9** |  |  | **9** |  |  | **10** |
| **1** | **5** | **12** | **9** | **9** |  |  | **9** |  |  | **9.75** |
| **2** | **0** | **10** | **11** | **10** | **10** | **11** | **6** |  |  | **10** |
| **2** | **1** | **10** | **11** | **10** | **10** | **11** | **6** |  |  | **10.5** |
| **2** | **2** | **10** | **11** | **10** | **10** | **11** | **6** |  |  | **10.33333** |
| **2** | **3** | **10** | **11** | **10** | **10** | **11** | **6** |  |  | **10.25** |
| **2** | **4** | **10** | **11** | **10** | **10** | **11** | **6** |  |  | **10.4** |
| **2** | **5** | **10** | **11** | **10** | **10** | **11** | **6** |  |  | **9.666667** |
| **3** | **0** | **11** | **12** | **11** | **11** | **10** | **8** |  |  | **11** |
| **3** | **1** | **11** | **12** | **11** | **11** | **10** | **8** |  |  | **11.5** |
| **3** | **2** | **11** | **12** | **11** | **11** | **10** | **8** |  |  | **11.33333** |
| **3** | **3** | **11** | **12** | **11** | **11** | **10** | **8** |  |  | **11.25** |
| **3** | **4** | **11** | **12** | **11** | **11** | **10** | **8** |  |  | **11** |
| **3** | **5** | **11** | **12** | **11** | **11** | **10** | **8** |  |  | **10.5** |
| **4** | **0** | **11** | **10** | **11** | **11** | **11** | **8** |  |  | **11** |
| **4** | **1** | **11** | **10** | **11** | **11** | **11** | **8** |  |  | **10.5** |
| **4** | **2** | **11** | **10** | **11** | **11** | **11** | **8** |  |  | **10.66667** |
| **4** | **3** | **11** | **10** | **11** | **11** | **11** | **8** |  |  | **10.75** |
| **4** | **4** | **11** | **10** | **11** | **11** | **11** | **8** |  |  | **10.8** |
| **4** | **5** | **11** | **10** | **11** | **11** | **11** | **8** |  |  | **10.33333** |
| **5** | **0** | **5** | **10** | **10** | **10** | **10** | **8** | **11** |  | **5** |
| **5** | **1** | **5** | **10** | **10** | **10** | **10** | **8** | **11** |  | **7.5** |
| **5** | **2** | **5** | **10** | **10** | **10** | **10** | **8** | **11** |  | **8.333333** |
| **5** | **3** | **5** | **10** | **10** | **10** | **10** | **8** | **11** |  | **8.75** |
| **5** | **4** | **5** | **10** | **10** | **10** | **10** | **8** | **11** |  | **9** |
| **5** | **5** | **5** | **10** | **10** | **10** | **10** | **8** | **11** |  | **8.833333** |
| **5** | **6** | **5** | **10** | **10** | **10** | **10** | **8** | **11** |  | **9.142858** |
| **6** | **0** | **7** | **10** | **9** | **12** | **12** | **12** | **12** | **10** | **7** |
| **6** | **1** | **7** | **10** | **9** | **12** | **12** | **12** | **12** | **10** | **8.5** |
| **6** | **2** | **7** | **10** | **9** | **12** | **12** | **12** | **12** | **10** | **8.666667** |
| **6** | **3** | **7** | **10** | **9** | **12** | **12** | **12** | **12** | **10** | **9.5** |
| **6** | **4** | **7** | **10** | **9** | **12** | **12** | **12** | **12** | **10** | **10** |
| **6** | **5** | **7** | **10** | **9** | **12** | **12** | **12** | **12** | **10** | **10.33333** |
| **6** | **6** | **7** | **10** | **9** | **12** | **12** | **12** | **12** | **10** | **10.57143** |
| **6** | **7** | **7** | **10** | **9** | **12** | **12** | **12** | **12** | **10** | **10.5** |
